# Neighborhood Deprivation, Race and Ethnicity, and Prostate Cancer Outcomes Across California Health Care Systems

**DOI:** 10.1001/jamanetworkopen.2024.2852

**Published:** 2024-03-19

**Authors:** Ananta Wadhwa, Charlotte Roscoe, Elizabeth A. Duran, Lorna Kwan, Candace L. Haroldsen, Jeremy B. Shelton, Jennifer Cullen, Beatrice S. Knudsen, Mathew B. Rettig, Saiju Pyarajan, Nicholas G. Nickols, Kara N. Maxwell, Kosj Yamoah, Brent S. Rose, Timothy R. Rebbeck, Hari S. Iyer, Isla P. Garraway

**Affiliations:** 1 Department of Surgical and Perioperative Care, Veterans Affairs (VA) Greater Los Angeles Healthcare System, Los Angeles, California; 2 Channing Division of Network Medicine, Department of Medicine, Brigham and Women’s Hospital, Boston, Massachusetts; 3 Department of Environmental Health, Harvard T. H. Chan School of Public Health, Boston, Massachusetts; 4 VA San Diego Healthcare System, San Diego, California; 5 Department of Radiation Oncology, University of California, San Diego, San Diego; 6 Center for Health Equity Education and Research, University of California, San Diego, La Jolla; 7 Department of Urology, David Geffen School of Medicine at UCLA (University of California, Los Angeles), Los Angeles; 8 Department of Internal Medicine, Division of Epidemiology, University of Utah, Salt Lake City; 9 IDEAS Center (COIN), VA Salt Lake City Healthcare System, Salt Lake City, Utah; 10 Department of Population and Quantitative Health Sciences, Case Western Reserve, Cleveland, Ohio; 11 Department of Medicine, Division of Hematology-Oncology, David Geffen School of Medicine at UCLA, Los Angeles; 12 UCLA Jonsson Comprehensive Cancer Center, Los Angeles; 13 VA Boston Healthcare System, Boston, Massachusetts; 14 Division of Hematology/Oncology, Department of Medicine, Perelman School of Medicine at the University of Pennsylvania, Philadelphia; 15 Department of Medicine, Corporal Michael J. Crescenz VA Medical Center, Philadelphia, Pennsylvania; 16 Department of Genetics, Perelman School of Medicine at the University of Pennsylvania, Philadelphia; 17 Department of Radiation Oncology, H. Lee Moffitt Cancer Center & Research Institute, Tampa, Florida; 18 James A. Haley Veterans Hospital, Tampa, Florida; 19 Department of Epidemiology, Harvard T. H. Chan School of Public Health, Boston, Massachusetts; 20 Department of Radiation Oncology, Dana-Farber Cancer Institute, Boston, Massachusetts; 21 Section of Cancer Epidemiology and Health Outcomes, Rutgers Cancer Institute of New Jersey, New Brunswick

## Abstract

**Importance:**

Non-Hispanic Black (hereafter, Black) individuals experience worse prostate cancer outcomes due to socioeconomic and racial inequities of access to care. Few studies have empirically evaluated these disparities across different health care systems.

**Objective:**

To describe the racial and ethnic and neighborhood socioeconomic status (nSES) disparities among residents of the same communities who receive prostate cancer care in the US Department of Veterans Affairs (VA) health care system vs other settings.

**Design, Setting, and Participants:**

This cohort study obtained data from the VA Central Cancer Registry for veterans with prostate cancer who received care within the VA Greater Los Angeles Healthcare System (VA cohort) and from the California Cancer Registry (CCR) for nonveterans who received care outside the VA setting (CCR cohort). The cohorts consisted of all males with incident prostate cancer who were living within the same US Census tracts. These individuals received care between 2000 and 2018 and were followed up until death from any cause or censoring on December 31, 2018. Data analyses were conducted between September 2022 and December 2023.

**Exposures:**

Health care setting, self-identified race and ethnicity (SIRE), and nSES.

**Main Outcomes and Measures:**

The primary outcome was all-cause mortality (ACM). Cox proportional hazards regression models were used to estimate hazard ratios for associations of SIRE and nSES with prostate cancer outcomes in the VA and CCR cohorts.

**Results:**

Included in the analysis were 49 461 males with prostate cancer. Of these, 1881 males were in the VA cohort (mean [SD] age, 65.3 [7.7] years; 833 Black individuals [44.3%], 694 non-Hispanic White [hereafter, White] individuals [36.9%], and 354 individuals [18.8%] of other or unknown race). A total of 47 580 individuals were in the CCR cohort (mean [SD] age, 67.0 [9.6] years; 8183 Black individuals [17.2%], 26 206 White individuals [55.1%], and 13 191 individuals [27.8%] of other or unknown race). In the VA cohort, there were no racial disparities observed for metastasis, ACM, or prostate cancer–specific mortality (PCSM). However, in the CCR cohort, the racial disparities were observed for metastasis (adjusted odds ratio [AOR], 1.36; 95% CI, 1.22-1.52), ACM (adjusted hazard ratio [AHR], 1.13; 95% CI, 1.04-1.24), and PCSM (AHR, 1.15; 95% CI, 1.05-1.25). Heterogeneity was observed for the racial disparity in ACM in the VA vs CCR cohorts (AHR, 0.90 [95% CI, 0.76-1.06] vs 1.13 [95% CI, 1.04-1.24]; *P* = .01). No evidence of nSES disparities was observed for any prostate cancer outcomes in the VA cohort. However, in the CCR cohort, heterogeneity was observed for nSES disparities with ACM (AHR, 0.82; 95% CI, 0.80-0.84; *P* = .002) and PCSM (AHR, 0.86; 95% CI, 0.82-0.89; *P* = .007).

**Conclusions and Relevance:**

Results of this study suggest that racial and nSES disparities were wider among patients seeking care outside of the VA health care system. Health systems–related interventions that address access barriers may mitigate racial and socioeconomic disparities in prostate cancer.

## Key Points

**Question:**

Does the magnitude of racial and ethnic and neighborhood socioeconomic disparities vary among individuals with prostate cancer residing in the same neighborhoods but seeking care in different health care systems?

**Findings:**

In this cohort study of 49 461 patients with prostate cancer, higher neighborhood deprivation was associated with worse survival in patients who received care in the community health care system compared with the relatively equal-access US Department of Veterans Affairs (VA) system. The racial disparities in all-cause mortality were significantly wider in the community health care population compared with the VA population.

**Meaning:**

Findings of this study suggest that interventions targeting access barriers experienced by patients with prostate cancer with lower socioeconomic status may mitigate neighborhood socioeconomic and racial disparities.

## Teaser

This cohort study compares all-cause mortality by neighborhood deprivation and race and ethnicity among individuals with prostate cancer receiving care in the US Department of Veterans Affairs (VA) health care system vs those receiving care outside the VA.

## Introduction

Self-identified race and ethnicity (SIRE), sociodemographic, and geographic disparities in prostate cancer outcomes are well documented.^1,2,3^ Non-Hispanic Black (hereafter, Black) compared with non-Hispanic White (hereafter, White) individuals have approximately 70% higher incidence and nearly double the mortality of prostate cancer.^1^ Complex and interacting multilevel factors, including structural racism, socioeconomic status or social determinants of health, environmental exposures, and heritable genetic variations, are associated with racial disparities.^4,5,6,7,8^ Numerous studies suggest that inequitable access to health care and related variables play a role in adverse prostate cancer outcomes^9,10,11^ but that receipt of care in the relatively equal-access US Department of Veterans Affairs (VA) health care system may be associated with the diminished roles of both race and rurality in prostate cancer outcomes.^12,13^

Higher neighborhood deprivation is also associated with worse survival in multiple cancers, including prostate cancer.^3^ The aim of this study was to describe racial and neighborhood socioeconomic status (nSES) disparities among individuals residing in the same communities who received prostate cancer care in the VA health care system vs other settings. We further sought to evaluate the heterogeneity of disparities observed in the VA vs non-VA health care systems. We hypothesized that associations between low nSES and prostate cancer outcomes (similar to the association with race and ethnicity) may not be observed in the relatively equal-access VA health care system.

## Methods

### Data Sources and Study Population

This retrospective cohort study obtained data from the VA Central Cancer Registry and the population-based California Cancer Registry (CCR) to compare veterans with prostate cancer who received care within the VA Greater Los Angeles Healthcare System (hereafter, VA cohort) with nonveterans receiving care in community or non-VA settings (hereafter, CCR cohort). We filtered or matched the CCR cohort to retain only those residing in US Census tracts with 1 or more veterans, to reduce confounding of association between health care system and prostate cancer outcomes by neighborhood-level factors. Both cohorts included males who were diagnosed with incident prostate cancer between January 1, 2000, and December 31, 2018, and followed up until death or censoring on December 31, 2018. The VA Greater Los Angeles Healthcare System Institutional Review Board (IRB) and the Research and Development Committee approved this cohort study for the VA cohort. The Dana-Farber Cancer Institute and the Rutgers Cancer Institute of New Jersey IRBs approved the study for the CCR cohort. All IRBs waived the informed consent requirement and Health Insurance Portability and Accountability Act of 1996 authorization because the study presented minimal risk, involving no patient contact, and because contacting all individuals would be prohibitively costly. We followed the Strengthening the Reporting of Observational Studies in Epidemiology (STROBE) reporting guideline.

The VA cohort was constructed using data from the VA Central Cancer Registry, accessed through the VA Informatics and Computing Infrastructure, as previously described.^12^ In addition, data were manually abstracted from the VA electronic health record (EHR), as previously described.^14^ Veterans with equivocal metastasis stage at diagnosis (n = 49) or missing spatial data (n = 159) were excluded (eFigure 1A in Supplement 1).

For CCR data, we excluded individuals who reported VA insurance (n = 45), had missing clinical stage data (n = 5257), or had missing spatial data (n = 100) (eFigure 1B in Supplement 1). Further details about the CCR cohort and matching procedures are provided in the eMethods in Supplement 1.

### Measures

Variables abstracted from the VA EHR included age at diagnosis; SIRE indicated on entry into the VA health care system; clinical tumor, lymph node, and metastasis stage at diagnosis and last follow-up; prostate cancer grade group; vital status (living, death due to prostate cancer, or death due to other causes); date of last follow-up; geographic coordinates; and income level. All clinical end points were confirmed through manual medical record review. In the CCR, sociodemographic and clinical factors were reported by facilities using medical records. To protect confidentiality, CCR officials geo-masked the CCR cohort's address geocodes using random displacement within 400 m of their residential address.^15^

To estimate nSES disparities at diagnosis and to align the socioeconomic disparity measure with the geographic scale of the study, we used a Census tract–level nSES index.^16^ We calculated a score by *z*-scaling each measure and reversing the scale for poverty and wealth measures so that increasing values indicated greater advantage. The score included median household income, median home value, percentage with a college degree, percentage of White residents, percentage of Black residents, percentage of foreign-born residents, percentage of families receiving interest or dividends, percentage of occupied housing units, and percentage of unemployed residents. To temporally align socioeconomic measures to years of diagnosis, we calculated scores separately using data from the 2000 Decennial Census (for individuals diagnosed between 2000 and 2005) and the 2006 to 2010 American Community Survey (for individuals diagnosed in 2006 or after). We parameterized the nSES index using a continuous term scaled to the IQR so that the effect estimate could be interpreted as the change in outcome associated with a 1-IQR increase in nSES. Quintiles were based on state-level cutoffs for the 2000 Decennial Census and the 2006 to 2010 American Community Survey.

### End Points

Prostate cancer incidence was identified from the use of *International Statistical Classification of Diseases and Related Health Problems, Tenth Revision (ICD-10)* codes. The primary end point was all-cause mortality (ACM). Secondary end points were de novo metastasis and prostate cancer–specific mortality (PCSM) assigned via manual medical record review (VA cohort) or use of the National Death Index (CCR cohort). The metastasis stage at diagnosis was defined as either no metastases detected or clinical evidence of metastases at the time of prostate cancer diagnosis and was identified through manual medical record review. Three VA-approved abstractors were trained in the VA EHR abstraction using an IRB-approved study abstraction tool. This tool has explicitly defined variables indicating metastasis at diagnosis of prostate cancer annotated in radiographic imaging reports and confirmed in the Central Cancer Registry notes and/or progress notes from urologic oncology, radiation oncology, and/or medical oncology practitioners. All metastatic annotations by abstractors were reviewed by supervising physicians (I.P.G., N.G.N.) to confirm interrater agreement or to resolve disagreement between raters.

### Statistical Analysis

Due to data use restrictions, we were unable to analyze a pooled database of individuals in both the VA and CCR cohorts; thus, we reported results separately by cohort. Kaplan-Meier analysis with log-rank tests were used to compare bivariate associations of SIRE and nSES with ACM. Multivariable logistic regression (de novo metastasis), Cox proportional hazards regression models (ACM), and Fine-Gray competing risk models (PCSM) were used to estimate odds ratios (ORs) and hazard ratios (HRs) for the associations of SIRE and nSES quintile with each outcome.

Models were adjusted for age at diagnosis, SIRE (Black, White, and other [Asian or Pacific Islander and Hispanic] or unknown), year of diagnosis, nSES (quintiles, Census tract level), stage at diagnosis (mortality end points only), and Census tract–level population density (quadratic). To evaluate heterogeneity in disparity measures estimated in the VA cohort compared with the CCR cohort, we calculated the Q statistic with 1 degree of freedom for the associations of continuous nSES and SIRE with each outcome.^17^ All tests were 2-sided with α = .05.

We performed several sensitivity analyses to assess the robustness of the findings to analytic decisions. E values were calculated to determine the magnitude of unmeasured confounding (eg, patient-level or environmental-level factor) required to attenuate the associations of nSES and SIRE to the null in the CCR cohort. In addition, we performed a fully matched cohort analysis, retaining the CCR cohort in strata of age, race and ethnicity, stage at diagnosis, year of diagnosis, and Census tract observed in the VA cohort. Further details are provided in the eMethods in Supplement 1.

Two-sided *P* < .05 indicated statistical significance. Data analysis was performed between September 2022 and December 2023 using SAS, version 9.4 (SAS Institute Inc), and R, version 4.3.1 (R Core Team).

## Results

### Study Population Characteristics

The study included 49 461 males with prostate cancer. Baseline characteristics of the VA (n = 1881) and CCR (n = 47 580) cohorts are summarized in Table 1. The VA cohort included 833 Black individuals (44.3%), 694 White individuals (36.9%), and 354 individuals (18.8%) of other or unknown race. The CCR cohort included 8183 Black individuals (17.2%), 26 206 White individuals (55.1%), and 13 191 individuals (27.8%) of other or unknown race. The VA cohort, compared with the CCR cohort, was younger (mean [SD] age, 65.3 [7.7] years vs 67.0 [9.6] years), more likely to self-identify as Black individuals (44.3% vs 17.2%), and more likely to be diagnosed with localized prostate cancer (94.6% vs 78.5%). Patients in the VA cohort were less likely than patients in the CCR cohort to reside in the most deprived nSES quintile (14.9% vs 21.4%). In both cohorts, Black patients were diagnosed at younger ages and were more likely to reside in urban Census tracts and in Census tracts with greater nSES disadvantage than their White counterparts (eTables 1 and 2 in Supplement 1).

**Table 1.** Characteristics of the Study Populations by Cohort

Characteristic	Cohort, No. (%)^a^	
	VA	CCR
No. of patients	1881	47 580
Age at diagnosis, mean (SD)	65.3 (7.7)	67.0 (9.6)
Age category, y		
≤50	42 (2.2)	1936 (4.1)
51-60	475 (25.3)	10 255 (21.6)
61-70	933 (49.6)	18 551 (39.0)
71-79	365 (19.4)	12 741 (26.8)
≥80	66 (3.5)	4097 (8.6)
SIRE^b^		
Black	833 (44.3)	8183 (17.2)
White	694 (36.9)	26 206 (55.1)
Other and unknown^c^	354 (18.8)	13 191 (27.8)
Year of diagnosis		
2000-2004	460 (24.5)	17 543 (36.9)
2005-2009	640 (34.0)	12 464 (26.2)
2010-2015	669 (35.6)	11 561 (24.3)
2016-2018	112 (6.0)	6012 (12.6)
Stage at diagnosis		
Localized	1780 (94.6)	37 356 (78.5)
Regional	57 (3.0)	7310 (15.4)
Distant	44 (2.3)	2914 (6.1)
Follow-up months, median (IQR)	120.00 (112.77-120.00)	89.62 (36.13-120.00)
Deaths from any cause	848 (45.1)	12 649 (26.6)
Prostate cancer diagnosis	113 (13.3)	3512 (7.4)
nSES index, mean (SD)	0.13 (3.57)	0.00 (3.46)
nSES quintile		
Q1 (most deprived)	280 (14.9)	10 167 (21.4)
Q2	415 (22.1)	9125 (19.2)
Q3	388 (20.6)	8193 (17.2)
Q4	387 (20.6)	9743 (20.5)
Q5 (least deprived)	411 (21.9)	10 352 (21.8)
Urbanicity: ≥1000 people/square mile	1675 (89.4)	41 114 (86.4)

Abbreviations: CCR, California Cancer Registry; nSES, neighborhood socioeconomic status; SIRE, self-identified race and ethnicity; VA, US Department of Veterans Affairs.

SI conversion factor: To convert miles to kilometers, multiply by 1.6.

^a^ Percentages may not sum to 100 due to rounding.

^b^ SIRE data were obtained from the Corporate Data Warehouse and CCR.

^c^ Other category included Asian or Pacific Islander and Hispanic.

**Figure 1.**
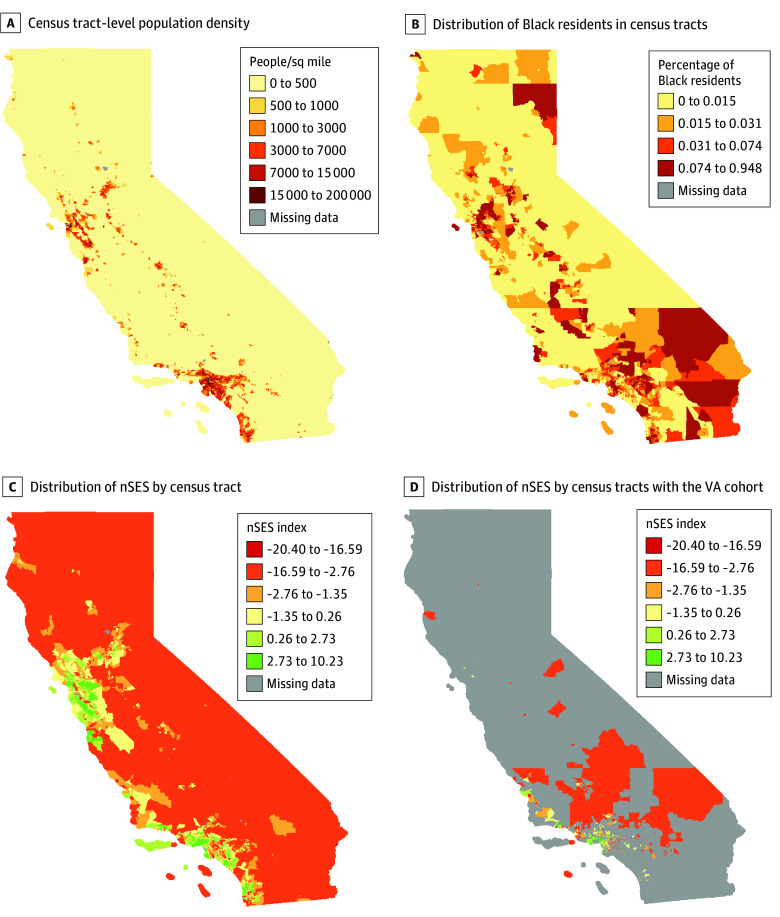
California Census Tract–Level Population Characteristics and Socioeconomic Factors in the Veterans Affairs (VA) and California Cancer Registry Cohorts nSES indicates neighborhood socioeconomic status.

Black individuals also were more likely to reside in coastal urban areas (Figure 1). The Census tracts in which the VA cohort resided were concentrated in Southern California. The distribution of Census tract socioeconomic measures was comparable between cohorts, but the VA cohort was more likely to reside in Census tracts with a higher percentage of Black residents compared with the CCR cohort (7.0% vs 3.1%) (eFigure 2 in Supplement 1).

### Racial and Socioeconomic Disparities in Survival

**Figure 2.**
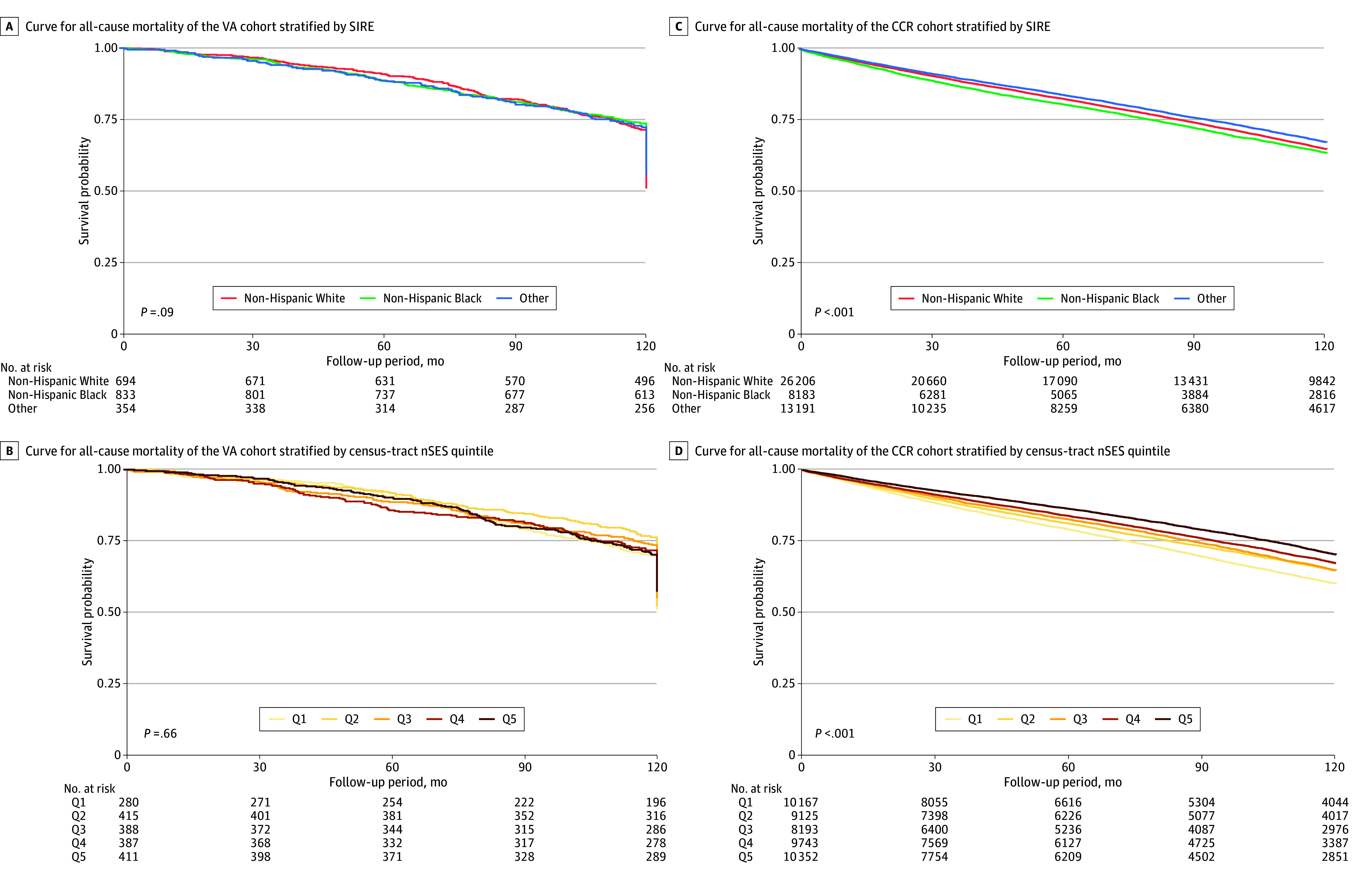
Kaplan-Meier Curves for All-Cause Mortality of the Veterans Affairs (VA) and California Cancer Registry (CCR) Cohorts nSES indicates neighborhood socioeconomic status; Q, quintile; SIRE, self-identified race and ethnicity.

Kaplan-Meier analysis showed that the 10-year all-cause survival was over 50% in both cohorts (Figure 2). There was no evidence of racial disparities in the VA cohort, but Black patients had poorer survival than those in other SIRE groups in the CCR cohort (*P* < .001). There was no evidence of socioeconomic disparities in the VA cohort, but there was a significant nSES gradient in survival in the CCR cohort (*P* < .001), with those in the least advantaged quintile of nSES having the lowest survival.

In adjusted models, there were no racial disparities observed for de novo metastasis, ACM, or PCSM in the VA cohort (Table 2), although point estimates indicated greater odds of de novo metastasis (adjusted OR [AOR], 1.19; 95% CI, 0.72-1.96) and higher risk of PCSM (adjusted HR [AHR], 1.13; 95% CI, 0.71-1.80) among Black compared with White patients. In the CCR cohort, Black patients had significantly higher odds of metastatic disease (AOR, 1.36; 95% CI, 1.22-1.52), and higher risk of ACM (AHR, 1.13; 95% CI, 1.04-1.24), and PCSM (AHR, 1.15; 95% CI, 1.05-1.25). In addition, patients in the other SIRE group had higher odds of de novo metastasis (AOR, 1.03; 95% CI, 1.00-1.21) but a lower risk of ACM (AHR, 0.83; 95% CI, 0.77-0.90) and PCSM (AHR, 0.84; 95% CI, 0.78-0.91). There was heterogeneity in the racial disparity for ACM observed in the VA cohort compared with the CCR cohort (AHR, 0.90 [95% CI, 0.76-1.06] vs 1.13 [95% CI, 1.04-1.24]; *P* = .01), but no evidence of heterogeneity was found comparing racial disparities for other end points between the 2 cohorts.

**Table 2.** Multivariable Analysis of Associations Between Self-Identified Race and Ethnicity and Prostate Cancer Outcomes in Both Cohorts^a^

Prostate cancer outcomes	White	Black	*P* value^b^	Other	*P* value^b^
**VA cohort**					
Advanced/localized, No.	35/659	47/786	NA	19/335	NA
De novo metastasis, AOR (95% CI)^c^	1 [Reference]	1.19 (0.72-1.96)	NA	1.07 (0.59-1.92)	NA
Deaths/person-years, No.	340/6179	351/7357	NA	157/3120	NA
ACM, AHR (95% CI)^d^	1 [Reference]	0.90 (0.76-1.06)	NA	0.85 (0.70-1.03)	NA
Deaths/person-years, No.	35/6179	54/7357	NA	24/3120	NA
PCSM, AHR (95% CI)^e^	1 [Reference]	1.13 (0.71-1.80)	NA	1.36 (0.79-2.34)	NA
**CCR cohort **					
Advanced/localized, No.	5636/20 570	1795/6388	NA	2793/10 398	NA
De novo metastasis, AOR (95% CI)^c^	1 [Reference]	1.36 (1.22-1.52)	.38	1.03 (1.00-1.21)	.65
Deaths/person-years, No.	7168/171 581	2296/51 316	NA	3185/83 550	NA
ACM, AHR (95% CI)^d^	1 [Reference]	1.13 (1.04-1.24)	.01	0.83 (0.77-0.90)	.33
Deaths/person-years, No.	1886/171 581	738/51 316	NA	888/83 550	NA
PCSM, AHR (95% CI)^e^	1 [Reference]	1.15 (1.05-1.25)	.99	0.84 (0.78-0.91)	.08

Abbreviations: ACM, all-cause mortality; AHR, adjusted hazard ratio; AOR, adjusted odds ratio; CCR, California Cancer Registry; NA, not applicable; nSES, neighborhood socioeconomic status; PCSM, prostate cancer–specific mortality; VA, US Department of Veterans Affairs.

^a^ All analyses included the covariates of race, nSES at the Census tract level (quintiles), age at diagnosis, year of diagnosis, and urbanicity. Models for mortality further included stage at diagnosis.

^b^ *P* value from Q Statistic with 1 degree of freedom comparing the heterogeneity of association in the CCR cohort to the VA cohort.

^c^ Calculated using multivariable logistic regression analysis.

^d^ Calculated using Cox proportional hazards regression model.

^e^ Calculated using Fine-Gray analysis for PCSM.

Disparities by nSES in the VA cohort compared with the CCR cohort are described in Table 3. We found no associations between nSES and de novo metastasis (AOR, 0.90; 95% CI, 0.72-1.13), ACM (AHR, 0.95; 95% CI, 0.87-1.04), or PCSM (AHR, 1.23; 95% CI, 0.95-1.58), although point estimates suggested higher risk of PCSM was associated with higher nSES. In the CCR cohort, no association was observed between nSES and de novo metastasis (AOR per IQR, 1.01; 95% CI, 0.99-1.04), but higher nSES was associated with lower risk of ACM (AHR, 0.82; 95% CI, 0.80-0.84; *P* = .002) and PCSM (AHR, 0.86; 95% CI, 0.82-0.89; *P* = .007). We observed heterogeneity in effect estimates obtained from the VA cohort compared with the CCR cohort for ACM and PCSM, indicating greater disparities observed in the CCR cohort vs the VA cohort.

**Table 3.** Multivariable Analysis of Associations Between Neighborhood Socioeconomic Status (nSES) and Prostate Cancer Outcomes in Both Cohorts^a^

	Continuous term scaled to IQR^a^	nSES Quintile 1	nSES Quintile 2	nSES Quintile 3	nSES Quintile 4	nSES Quintile 5	*P* for trend	*P* value^b^
**VA cohort**								
Advanced/localized, No.	NA	15/265	19/396	20/68	27/360	20/391	NA	NA
De novo metastasis, AOR (95% CI)^c^	0.90 (0.72-1.13)	1 [Reference]	1.14 (0.55-2.36)	1.01 (0.50-2.05)	1.25 (0.64-2.44)	0.74 (0.37,1.51)	.31	NA
Deaths/person-years, No.	NA	137/2471	198/3751	174/3422	164/3378	175/3631	NA	NA
ACM, AHR (95% CI)^d^	0.95 (0.87-1.04)	1 [Reference]	0.88 (0.71-1.10)	0.89 (0.71-1.12)	0.83 (0.66-1.04)	0.87 (0.70-1.10)	.24	NA
Deaths/person-years, No.	NA	14/2471	31/3751	19/3422	28/3378	21/3631	NA	NA
PCSM, AHR (95% CI)^e^	1.23 (0.95-1.58)	1 [Reference]	1.70 (0.89-3.26)	0.98 (1.02-0.48)	1.51 (0.78-2.91)	1.46 (0.73-2.93)	.50	NA
**CCR cohort **								
Advanced/localized, No.	NA	1986/8181	1913/7212	1779/6414	2147/7596	2399/7953	NA	NA
De novo metastasis, AOR (95% CI)^c^	1.01 (0.99-1.04)	1 [Reference]	1.08 (1.01-1.17)	1.06 (0.99-1.14)	1.06 (0.99-1.14)	1.07 (1.00-1.15)	.16	.33
Deaths/person-years, No.	NA	3339/67 152	2694/62 736	2203/52 751	2364/61 850	2049/61 959	NA	NA
ACM, AHR (95% CI)^d^	0.82 (0.80-0.84)	1 [Reference]	0.88 (0.84-0.93)	0.83 (0.78-0.87)	0.74 (0.71-0.79)	0.63 (0.60-0.67)	<.001	.002
Deaths/person-years, No.	NA	906/67 152	741/62 736	637/52 751	642/61 850	586/61 959	NA	NA
PCSM, AHR (95% CI)^e^	0.86 (0.82-0.89)	1 [Reference]	0.90 (0.82-0.99)	0.90 (0.81-0.99)	0.79 (0.71-0.87)	0.72 (0.65-0.79)	<.001	.007

Abbreviations: ACM, all-cause mortality; AHR, adjusted hazard ratio; AOR, adjusted odds ratio; CCR, California Cancer Registry; NA, not applicable; PCSM, prostate cancer–specific mortality; VA, US Department of Veterans Affairs.

^a^ All analyses included the covariates of race, nSES at the Census tract level (quintiles), age at diagnosis, year of diagnosis, and urbanicity. Models for mortality further included stage at diagnosis.

^b^ *P* value from Q Statistic with 1 degree of freedom comparing heterogeneity of association in the CCR cohort to the VA cohort.

^c^ Calculated using multivariable logistic regression analysis.

^d^ Calculated using Cox proportional hazards regression model.

^e^ Calculated using Fine-Gray analysis for PCSM.

### Sensitivity Analysis

Results of sensitivity analyses for unmeasured confounding are presented in eTable 3 in Supplement 1. E-values report the magnitude of the association between an unmeasured confounder and either the exposure (nSES or SIRE) or the outcome (de novo metastasis, ACM, or PCSM). In a comparison of quintile 5 to quintile 1 of nSES, the E-values for the CI ranged from 1.96 for ACM to 1.64 for PCSM, suggesting that an omitted variable would have to exhibit a more robust independent association with either nSES or prostate cancer outcomes than most known prostate cancer risk factors to shift the CI to include 1. In a comparison of prostate cancer outcomes between Black and White patients, the E-values for CIs ranged from 1.20 to 1.44.

Characteristics of the fully matched CCR cohort (n = 2914) stratified by SIRE are presented in eTable 4 in Supplement 1. In the fully matched CCR cohort, disparity estimates were less precise, but point estimates indicated higher ACM and PCSM in Black compared with White patients (eTable 5 in Supplement 1). There was no evidence of heterogeneity in racial disparity estimates for those in the Black or other SIRE groups compared with White patients across cohorts. Higher nSES was associated with higher odds of advanced stage at diagnosis (AOR per IQR increase: 1.11; 95% CI, 0.99-1.25), but lower risks of ACM (AHR, 0.78; 95% CI, 0.71-0.86) and PCSM (AHR, 0.91; 95% CI, 0.72-1.13), although only the association of ACM reached significance in the fully matched CCR cohort (eTable 6 in Supplement 1). There was heterogeneity in the association between nSES and PCSM in the VA cohort compared with the fully matched CCR cohort (AHR, 1.23 [95% CI, 0.95-1.58] vs 0.91 [95% CI, 0.72-1.13]; *P* = .005), but not for other outcomes.

## Discussion

We found significant heterogeneity in the magnitude of racial and neighborhood socioeconomic disparities in ACM and PCSM between veterans with prostate cancer who received care in the relatively equal-access VA Greater Los Angeles Healthcare System and nonveterans residing in the same neighborhoods but who sought care outside of the VA system. Racial disparities in prostate cancer outcomes were observed in the CCR cohort but not the VA cohort, although evidence of heterogeneity was observed only for ACM. For nSES disparities, heterogeneity across cohorts was observed, with robust evidence of association between higher ACM and PCSM and greater neighborhood deprivation observed in the CCR cohort but not in the VA cohort.

These findings are consistent with previous studies suggesting that inequities in health care access are important modifiable factors in racial disparities.^2,18,19,20^ By restricting the CCR cohort to individuals with the same residential Census tracts as the VA cohort, we limited the potential for neighborhood-level confounding that could be correlated with VA health care access and prostate cancer outcomes. Given the paucity of modifiable behavioral and environmental risk factors for aggressive and fatal prostate cancer,^21^ differences in patient-level characteristics are unlikely to explain the heterogeneity in prostate cancer disparities observed across the 2 cohorts. Therefore, the most likely explanation for observed differences in prostate cancer disparities is that health systems–level factors and quality of care are less variable across socioeconomic and racial and ethnic groups of patients treated in the VA system vs other settings. Moreover, this study suggests that the nSES index, which captures income, employment, educational, housing, and occupational factors, may have a more robust association with health care access for patients outside of the VA system.

Previous studies simulating the outcomes of equalizing access to care in diverse cohorts suggested that SIRE-based prostate cancer disparities would be substantially reduced or eliminated.^11^ By expanding health care access through minimizing financial barriers, the VA health care system may have successfully eliminated a socioeconomic gradient in outcomes for its patients. Veterans using the VA system likely benefit from additional services that decrease access barriers. For example, the VA has made national-level investments in infrastructure to provide guidance for management of prostate cancer, among other cancers. These investments include providing clinical pathways, akin to a national guideline, for management of prostate cancer and providing the most up-to-date, evidence-based prostate cancer treatments.^22^ Additionally, eligible veterans can receive short-term housing, transportation, service-connected income, and access to telehealth services, services that are rarely, if ever, accessible in other health care settings. It is also possible that since patients with prostate cancer require long-term follow-up, there is an opportunity to detect other medical issues and address them, which could play a role in decreased ACM in veterans with access to regular VA care. However, VA health care has well-known limitations, including a history of prolonged wait times for some services and lengthy travel for some veterans seeking to access VA facilities.^23^

Several recent studies highlight potential differences in the biological features of prostate cancer observed in Black and White individuals, and it is possible that more aggressive tumor biology, including ancestry-linked genetic alternations, may interact with SIRE, health systems, and sociopolitical systems.^8,24^ However, multilevel studies examining both genetics and social environment of cancer outcomes show more robust associations of tumor characteristics with incidence than with survival.^25,26,27,28^ Results of the present study join a growing body of work in demonstrating that a critical factor in SIRE-based survival disparities is the inequity in health care access.^2,9,10,19,20,29^ Health care access serves as an important modifiable pathway through which social inequities lead to health disparities. This study showed that reported racial and socioeconomic disparities were significantly attenuated among individuals seeking care in a more vs less equitable health care system, suggesting that health care access could be a means of eliminating disparities in prostate cancer.

### Limitations

Limitations of this study include the incomplete capture of specific VA care benefits that are absent in other health care systems but target inequities, which we argue may explain the associations between SIRE and nSES that were observed in the CCR cohort but not the VA cohort. There may be unmeasured behavioral, environmental, treatment, and access-related factors that vary across Black and White populations in the VA and CCR cohorts and may operate independently of the health care system; however, results of the sensitivity analyses suggested that confounding is unlikely to change our inference regarding the presence of disparities in ACM and PCSM observed in the CCR cohort. Cause-of-death ascertainment may have varied in the CCR vs the VA cohort, introducing misclassification bias. In the CCR cohort, we lacked data on health care systems (eg, academic medical center, federally qualified health care center). Geomasking may introduce nondifferential measurement error to the assessment of nSES, but it protects individual privacy.^30^ The study data did not allow for an investigation of tumor biological factors, such as molecular features, which may be associated with disparities. Additionally, this analysis involved a single VA institution (VA Greater Los Angeles Healthcare System) in California that is also a Prostate Cancer Center of Excellence, wherein a focus on standardized, evidence-based care protocols may be a factor in achieving equitable outcomes.^31^ Further studies are needed to assess the generalizability of these results to other states.

While these limitations are important, many of them can be addressed in future studies evaluating the interactions among SIRE, nSES, and prostate cancer outcomes. The study findings suggest that health systems such as the VA that promote equitable health care access (and possibly quality of care) may be effective in narrowing racial and socioeconomic disparities in prostate cancer. Along with continued research on variation in racial and socioeconomic disparities across different patient groups and health care settings, greater efforts are needed to promote equitable access to care for prostate cancer (and other cancers) and align stakeholder incentives toward these goals in the US and globally.^32^

## Conclusions

Among individuals with prostate cancer residing in the same Census tracts but receiving care in different health systems, racial and neighborhood socioeconomic disparities had a greater magnitude among individuals who sought care in health care systems with less equal access. Integrating molecular and tumor biological factors, along with health care systems and prostate cancer outcomes, could further clarify the multilevel factors associated with prostate cancer disparities. These analyses should be replicated across a wider range of geographic locations, with more detailed patient-level and environmental factors to confirm the findings.

## Notes

Supplement 1. **eMethods.** Description of California Cancer Registry Matched Design, Sensitivity Analyses **eTable 1.** Characteristics of the Greater Los Angeles Veterans Administration Study Population Stratified by Race and Ethnicity (n=1,881) **eTable 2.** Characteristics of the California Cancer Registry Census Tract Matched Study Population Stratified by Race and Ethnicity (n=47,580) **eTable 3.** E-Values for Odds Ratio and Hazard Ratio Estimates of Associations Between Neighborhood Socioeconomic Status and Non-Hispanic Black Race and Prostate Cancer Outcomes in the Geographically Matched California Cancer Registry Cohort **eTable 4.** Characteristics of the California Cancer Registry Cohort Matched to Greater Los Angeles Veterans Health Administration on Age, Race, Diagnosis Year, Stage, and Census Tract Stratified by Race and Ethnicity (n=2,914) **eTable 5.** Multivariable Analysis for Associations of Neighborhood Socioeconomic Status With Prostate Cancer Outcomes in the Veterans Health Administration Cohort and Fully Matched (Age, Race, Diagnosis Year, Stage, Census Tract) California Cancer Registry Cohort **eTable 6.** Multivariable Analysis for Associations of Race and Ethnicity With Prostate Cancer Outcomes in the Veterans Health Administration Cohort and Fully Matched (Age, Race, Diagnosis Year, Stage, Census Tract) California Cancer Registry Cohort **eFigure 1.** Study Flow Diagrams for (A) Greater Los Angeles Veterans Administration and (BC) California Cancer Registry Census Tract-Matched Cohorts **eFigure 2.** Distribution of Census Tract Socioeconomic Measures in California Compared to the Census Tracts in the Greater Los Angeles Veterans Health Administration

Supplement 2.
Data Sharing Statement
