# Supplementary material for: Neighborhood Deprivation, Race and Ethnicity, and Prostate Cancer Outcomes Across California Health Care Systems
Source: JAMA Netw Open. 2024 Mar 19;7(3):e242852. doi: 10.1001/jamanetworkopen.2024.2852 (PMC10951732; doi:10.1001/jamanetworkopen.2024.2852)
Supplement: Supplement 1. — eMethods. Description of California Cancer Registry Matched Design, Sensitivity Analyses eTable 1. Characteristics of the Greater Los Angeles Veterans Administration Study Population Stratified by Race and Ethnicity (n=1,881) eTable 2. Characteristics of the California Cancer Registry Census Tract Matched Study Population Stratified by Race and Ethnicity (n=47,580) eTable 3. E-Values for Odds Ratio and Hazard Ratio Estimates of Associations Between Neighborhood Socioeconomic Status and Non-Hispanic Black Race and Prostate Cancer Outcomes in the Geographically Matched California Cancer Registry Cohort eTable 4. Characteristics of the California Cancer Registry Cohort Matched to Greater Los Angeles Veterans Health Administration on Age, Race, Diagnosis Year, Stage, and Census Tract Stratified by Race and Ethnicity (n=2,914) eTable 5. Multivariable Analysis for Associations of Neighborhood Socioeconomic Status With Prostate Cancer Outcomes in the Veterans Health Administration Cohort and Fully Matched (Age, Race, Diagnosis Year, Stage, Census Tract) California Cancer Registry Cohort eTable 6. Multivariable Analysis for Associations of Race and Ethnicity With Prostate Cancer Outcomes in the Veterans Health Administration Cohort and Fully Matched (Age, Race, Diagnosis Year, Stage, Census Tract) California Cancer Registry Cohort eFigure 1. Study Flow Diagrams for (A) Greater Los Angeles Veterans Administration and (BC) California Cancer Registry Census Tract-Matched Cohorts eFigure 2. Distribution of Census Tract Socioeconomic Measures in California Compared to the Census Tracts in the Greater Los Angeles Veterans Health Administration [file jamanetwopen-e242852-s001.pdf]

Supplemental Online Content

Wadhwa A, Roscoe C, Duran EA, et al. Neighborhood deprivation, race and ethnicity, and prostate cancer outcomes across California health care systems. *JAMA Netw Open*. 2024;7(3):e242852. doi:10.1001/jamanetworkopen.2024.2852

eMethods. Description of California Cancer Registry Matched Design, Sensitivity Analyses

eTable 1. Characteristics of the Greater Los Angeles Veterans Administration Study Population Stratified by Race and Ethnicity (n=1,881)

eTable 2. Characteristics of the California Cancer Registry Census Tract Matched Study Population Stratified by Race and Ethnicity (n=47,580)

eTable 3. E-Values for Odds Ratio and Hazard Ratio Estimates of Associations Between Neighborhood Socioeconomic Status and Non-Hispanic Black Race and Prostate Cancer Outcomes in the Geographically Matched California Cancer Registry Cohort

eTable 4. Characteristics of the California Cancer Registry Cohort Matched to Greater Los Angeles Veterans Health Administration on Age, Race, Diagnosis Year, Stage, and Census Tract Stratified by Race and Ethnicity (n=2,914)

eTable 5. Multivariable Analysis for Associations of Neighborhood Socioeconomic Status With Prostate Cancer Outcomes in the Veterans Health Administration Cohort and Fully Matched (Age, Race, Diagnosis Year, Stage, Census Tract) California Cancer Registry Cohort

eTable 6. Multivariable Analysis for Associations of Race and Ethnicity With Prostate Cancer Outcomes in the Veterans Health Administration Cohort and Fully Matched (Age, Race, Diagnosis Year, Stage, Census Tract) California Cancer Registry Cohort

eFigure 1. Study Flow Diagrams for (A) Greater Los Angeles Veterans Administration and (BC) California Cancer Registry Census Tract-Matched Cohorts

eFigure 2. Distribution of Census Tract Socioeconomic Measures in California Compared to the Census Tracts in the Greater Los Angeles Veterans Health Administration

This supplemental material has been provided by the authors to give readers additional information about their work.

© 2024 Wadhwa A et al. *JAMA Network Open*

eMethods.

California Cancer Registry - Study population and design

To compare outcomes among men with prostate cancer (PCa) diagnosed in the Greater Los Angeles Veterans Affairs (VA) Healthcare System to those who resided in the same neighborhoods but sought care in other healthcare systems, we designed a complementary cohort using data from the population-based California Cancer Registry (CCR). All cases of cancer diagnosed in facilities, laboratories, and medical offices are required to be reported to the registry. Accreditation for the CCR is provided by the Surveillance, Epidemiology, and End Results (SEER) and the Centers for Disease Control and Prevention's National Program of Cancer Registries (NPCR), based on completeness and accuracy of reported data. Deaths are ascertained through linkages with the National Death Index, hospital, state records, and other sources. Cause of death from PCa was ascertained using ICD-09 (185) and ICD-10 (C61) codes. Both sets of codes were used due to a lag in adoption of ICD-10 codes.

We sought to match the design of the CCR cohort as closely as possible to the VA cohort. Men diagnosed with PCa between January 1, 2000, and December 31, 2018, were followed until death or censoring at the end of follow-up, whichever came first. Eligibility criteria included residence in census tracts where VA participants were residing, to match on similar neighborhood characteristics, including ADI, resulting in 52,837 eligible men. We excluded men whose insurance indicated care received at the VA (n=45), who were missing stage at diagnosis (n=5,112), and who were missing spatial identifiers (n=100), leading to a final sample of 47,580 men. The registry ascertains deaths annually through linkages with the National Death Index and state records.

Geomasking address-level geocodes to protect confidentiality

In order to protect confidentiality and abide by privacy regulations governing use of CCR data for research, CCR participant address geocodes at time of diagnosis were geomasked using a random spatial displacement[1,2]. This procedure involved setting a radius around each address geocode and randomly displacing the geocode from the origin based on a Gaussian distribution with 3 standard deviations set to 400m, which was deemed satisfactory for protecting confidentiality within the registry's guidelines. In addition, the direction (north or south for latitude, east or west for longitude) of displacement was assigned from a random Bernoulli distribution. This process was repeated for every observation in the database. While this procedure was different from the approach for geocoding in the VA, which used actual addresses, the spatial displacement was restricted to a 400m radius surrounding the participant's actual address, limiting potential misclassification of census tract. Evaluations comparing different geomasking approaches suggest that spatial displacement within a small geographic area provide an appropriate balance between privacy protection while preserving fidelity of exposure assessment[3].

Sensitivity analysis for confounding by measured demographic, clinical and geographic factors

As a sensitivity analysis to evaluate impact of confounding by study population characteristics that differ between the Greater Los Angeles Veterans Administration cohort and the California Cancer Registry population-based cohort, we conducted a refined matched cohort design. We identified strata of age (10-year categories), race and ethnicity (Non-Hispanic White, Non-Hispanic Black, Other), stage at diagnosis (Localized, Regional/Distant), Year of diagnosis (2000-2004, 2005-2009, 2010-2015, 2016-2018), and census tract in the Greater Los Angeles Veterans Administration cohort of men with prostate cancer and used these strata to filter the California Cancer Registry population-based cohort. Only men in strata with ≥1 man with prostate cancer were retained. Following this matching procedure, there were

2,914 men with prostate cancer who remained in the same strata. We refer to this matched cohort as the "fully matched" cohort.

Statistical tests for heterogeneity in the Veterans Administration, California Cancer Registry Geographically Matched Cohort, and the California Cancer Registry Fully Matched Cohorts

Due to data use restrictions, we were unable to analyze a pooled database of participants from the Veterans Administration and the California Cancer Registry. Therefore, we performed tests of heterogeneity using the Q-statistic with 1 degree of freedom for linear associations between nSES and each outcome (de novo metastasis, all-cause mortality, prostate cancer-specific mortality)[4]. We separately compared these associations in the Greater Los Angeles Veterans Administration to the Geographically matched California Cancer Registry and the Fully matched California Cancer Registry cohorts. Tests were two-sided with alpha=0.05.

Sensitivity analysis for unmeasured confounding

We compared associations between nSES, SIRE and PCa outcomes in the VA vs CCR to test the hypothesis that the health system in which a patient receives care can mitigate PCa outcome disparities associated with Black/African American race and nSES. However, unmeasured confounding arising from differences in patient-level factors (e.g., clinical factors, individual socioeconomic status) between the VA and CCR populations might also explain why disparities associated with nSES and SIRE are observed in CCR but not the VA.

The E-value is a statistical measure that has been proposed to quantify the minimum strength of association required by an unmeasured confounder with either the exposure (in this case, either Black/African American race or ADI) or outcome (in this case, metastatic PCa at diagnosis, ACM, or PCSM) to either shift the point estimate to the null value, or shift the confidence interval bound closest to the null to contain the null value, conditional on measured covariates[5]. Because epidemiologic associations are often reported on the ratio scale, the E-value can be interpreted as an odds or risk ratio for the association between the unmeasured confounding variable and either the exposure or outcome. E-values are increasingly used in epidemiologic research to evaluate the robustness of reported associations to unmeasured confounding[6].

We calculated E-values for associations between Non-Hispanic Black/African American race, nSES and PCa outcomes in the CCR. Because outcomes were common, we did not apply the rare disease assumption when translating odds ratios and hazard ratios to E-values on the risk ratio scale. We then compared the magnitude of E-values for associations in CCR to the strength of associations with clinical factors reported in the literature.

References

1. Armstrong MP, Rushton G, Zimmerman DL. Geographically masking health data to preserve confidentiality. Stat Med. 1999 Mar 15;18(5):497–525.

2. Wang J, Kim J, Kwan MP. An exploratory assessment of the effectiveness of geomasking methods on privacy protection and analytical accuracy for individual-level geospatial data. Cartogr Geogr Inf Sci. 2022 Sep 3;49(5):385–406.

© 2024 Wadhwa A et al. *JAMA Network Open*

3. Iyer HS, Shi X, Satagopan JM, Cheng I, Roscoe C, McLaughlin RH, et al. Advancing Social and Environmental Research in Cancer Registries Using Geomasking for Address-Level Data. Cancer Epidemiol Biomarkers Prev. 2023 Nov 1;32(11):1485–9.

4. DerSimonian R, Laird N. Meta-analysis in clinical trials. Control Clin Trials. 1986 Sep;7(3):177–88.

5. VanderWeele TJ, Ding P. Sensitivity Analysis in Observational Research: Introducing the E-Value. Ann Intern Med. 2017 Aug 15;167(4):268–74.

6. Blum MR, Tan YJ, Ioannidis JPA. Use of E-values for addressing confounding in observational studies-an empirical assessment of the literature. Int J Epidemiol. 2020 Oct 1;49(5):1482–94.

Supplementary Results

E-values for unmeasured confounding

E-values for associations between NHB/AA and nSES are presented in **eTable 4**. The strongest E-values observed were for associations between nSES Quintile 5 vs 1 for ACM (Estimate, Confidence Interval: 2.10, 1.96) and PCSM (Estimate, Confidence Interval: 1.82, 1.64). E-values for associations between NHB/AA and odds of metastatic PCa (1.61, 1.44), ACM (1.40, 1.20), and PCSM (1.44, 1.22) were moderately strong. These E-Values suggest that an unmeasured confounding variable would need have an association with PCa outcomes on the risk ratio scale ranging from 1.20-1.96 to shift the confidence interval for associations of nSES or race and ethnicity with PCa outcomes to contain the null value of 1. It is plausible that unmeasured factors may exhibit relative risk ratios within the range of 1.20-1.96 with outcomes, but these factors (particularly related to access to care) would lie along the causal path between health care system where care is received and PCa outcomes.

These findings support our hypothesis that patients receiving care in the VA may receive health systems-level benefits that are associated with clinical characteristics at diagnosis and over follow-up, which then reduce the strength of associations of nSES and race and ethnicity with PCa outcomes. We interpret the "unmeasured confounding by clinical characteristics" seen in the CCR as evidence of differences in the healthcare system where patients in the VA seek care compared to the CCR, which translate to statistical differences in clinical presentation and outcomes over follow-up associated with race and nSES. The observation that confounding from unmeasured clinical factors may "explain" associations between race and nSES in the CCR provide support for the hypothesis that health systems factors that vary between the VA and other settings are a major contributor to observed racial disparities in PCa in population-based databases.

© 2024 Wadhwa A et al. *JAMA Network Open*

eTable 1. Characteristics of the Greater Los Angeles Veterans Administration Study Population Stratified by Race and Ethnicity (n=1,881)

	**Overall**	**NHW**	**NHB**	**Other**	***P*[a]**
**n**	**1,881**	**694 (36.9)**	**833 (44.3)**	**354 (18.8)**	
Age at diagnosis [mean (SD)]	65.27 (7.74)	65.71 (7.61)	64.35 (7.73)	66.62 (7.76)	<0.001
Age category (years, %)					<0.001
<50	42 (2.2)	<11[d]	25 (3.0)	<11[d]	
51-60	475 (25.3)	170 (24.5)	248 (29.8)	57 (16.1)	
61-70	933 (49.6)	342 (49.3)	400 (48.0)	191 (54.0)	
71-79	365 (19.4)	144 (20.7)	140 (16.8)	81 (22.9)	
80+	66 (3.5)	29 (4.2)	20 (2.4)	17 (4.8)	
Diagnosis years (%)					0.011
2000-2004	460 (24.5)	183 (26.4)	180 (21.6)	97 (27.4)	
2005-2009	640 (34.0)	252 (36.3)	269 (32.3)	119 (33.6)	
2010-2015	669 (35.6)	227 (32.7)	322 (38.7)	120 (33.9)	
2016-2018	112 (6.0)	32 (4.6)	62 (7.4)	18 (5.1)	
Stage at diagnosis (%)					0.687
Localized	1780 (94.6)	659 (95.0)	786 (94.4)	335 (94.6)	
Regional	57 (3.0)	23 (3.3)	25 (3.0)	<11[d]	
Distant	44 (2.3)	12 (1.7)	22 (2.6)	<11[d]	
Follow-up months (median [IQR])	120.00 [112.77, 120.00]	120.00 [112.68, 120.00]	120.00 [114.35, 120.00]	120.00 [111.83, 120.00]	0.867[b]
Deaths from any cause (%)	848 (45.1)	340 (49.0)	351 (42.1)	157 (44.4)	0.026
Prostate cancer (%)	113 (13.3)	35 (10.2)	54 (15.4)	24 (15.3)	0.397
nSES Index (mean (SD))	0.13 (3.57)	1.11 (3.66)	-0.67 (3.31)	0.11 (3.49)	<0.001
nSES Quintiles (%)					<0.001
Q1	280 (14.9)	81 (11.7)	142 (17.0)	57 (16.1)	
Q2	415 (22.1)	92 (13.3)	346 (29.5)	77 (21.8)	
Q3	388 (20.6)	130 (18.7)	180 (21.6)	78 (22.0)	
Q4	387 (20.6)	177 (25.5)	140 (16.8)	70 (19.8)	
Q5	411 (21.9)	214 (30.8)	125 (15.0)	72 (20.3)	
Urbanicity (Population density ≥1000 people/mi^2) (%)	1675 (89.4)	585 (84.4)	779 (94.2)	311 (87.9)	<0.001

[a]Chi-squared test of independence, [b]Wilcoxon Rank Sum Test, [c]Independent two-sample t-test, [d]Cell counts and percentages suppressed for confidentiality. (% or SD), [range]. Percentages may not sum to 100 due to rounding. Abbreviations: NHB = Non-Hispanic Black, NHW = Non-Hispanic White, nSES = Neighborhood socioeconomic status, IQR = interquartile range

© 2024 Wadhwa A et al. *JAMA Network Open*

eTable 2. Characteristics of the California Cancer Registry Census Tract Matched Study Population Stratified by Race and Ethnicity (n=47,580)

	Overall	NHW	NHB	Oth	*P*[a]
**n**	**47580**	**26206 (55%)**	**8183 (17%)**	**13191 (28%)**	
Age at diagnosis [mean (SD)]	67.02 (9.59)	67.62 (9.53)	64.49 (9.49)	67.38 (9.52)	<0.001[c]
Age category (years, %)					<0.001
<50	1936 (4.1)	873 (3.3)	575 (7.0)	488 (3.7)	
51-60	10255 (21.6)	5393 (20.6)	2216 (27.1)	2646 (20.1)	
61-70	18551 (39.0)	9981 (38.1)	3352 (41.0)	5218 (39.6)	
71-79	12741 (26.8)	7465 (28.5)	1607 (19.6)	3669 (27.8)	
80+	4097 (8.6)	2494 (9.5)	433 (5.3)	1170 (8.9)	
Diagnosis years (%)					<0.001
2000-2004	17543 (36.9)	10234 (39.1)	2871 (35.1)	4438 (33.6)	
2005-2009	12464 (26.2)	6818 (26.0)	2159 (26.4)	3487 (26.4)	
2010-2015	11561 (24.3)	6015 (23.0)	2108 (25.8)	3438 (26.1)	
2016-2018	6012 (12.6)	3139 (12.0)	1045 (12.8)	1828 (13.9)	
Stage at diagnosis (%)					<0.001
Localized	37356 (78.5)	20570 (78.5)	6388 (78.1)	10398 (78.8)	
Regional	7310 (15.4)	4224 (16.1)	1161 (14.2)	1925 (14.6)	
Distant	2914 (6.1)	1412 (5.4)	634 (7.7)	868 (6.6)	
Follow-up months (median [IQR])	89.62 [36.13, 120.00]	92.48 [37.71, 120.00]	84.89 [33.30, 120.00]	86.53 [34.78, 120.00]	<0.001[b]
Deaths from any cause (%)	12649 (26.6)	7168 (27.4)	2296 (28.1)	3185 (24.1)	<0.001
Prostate cancer (%)	3512 (7.4)	1886 (7.2)	738 (9.0)	888 (6.7)	<0.001
nSES Index (mean (SD))	0.00 (3.46)	0.64 (3.89)	-0.90 (2.51)	-0.72 (2.72)	<0.001
nSES Quintiles (%)					
Q1	10167 (21.4)	5032 (19.2)	2029 (24.8)	3106 (23.5)	
Q2	9125 (19.2)	3378 (12.9)	2444 (29.9)	3303 (25.0)	
Q3	8193 (17.2)	4099 (15.6)	1404 (17.2)	2690 (20.4)	
Q4	9743 (20.5)	5909 (22.5)	1384 (16.9)	2450 (18.6)	
Q5	10352 (21.8)	7788 (29.7)	922 (11.3)	1642 (12.4)	
Urbanicity (Population density ≥1000 people/mi^2) (%)	41114 (86.4)	21033 (80.3)	7888 (96.4)	12193 (92.4)	<0.001

[a]Chi-squared test of independence, [b]Wilcoxon Rank Sum Test, [c]Independent two-sample t-test, (% or SD), [range]. Percentages may not sum to 100 due to rounding. Abbreviations: NHB = Non-Hispanic Black, NHW = Non-Hispanic White, nSES = Neighborhood socioeconomic status, IQR = interquartile range

© 2024 Wadhwa A et al. *JAMA Network Open*

eTable 3. E-Values for Odds Ratio and Hazard Ratio Estimates of Associations between Neighborhood Socioeconomic Status and Non-Hispanic Black and Prostate Cancer Outcomes in the Geographically Matched California Cancer Registry Cohort

	Metastatic Prostate Cancer at Diagnosis	All-Cause Mortality	Prostate Cancer-Specific Mortality
**E-Value**	**Estimate, CI**	**Estimate, CI**	**Estimate, CI**
**nSES**			
Q1	1.00, Referent	1.00, Referent	1.00, Referent
Q2	1.24, 1.08	1.42, 1.30	1.36, 1.09
Q3	1.20, 1.00	1.53, 1.44	1.36, 1.09
Q4	1.20, 1.00	1.76, 1.64	1.64, 1.44
Q5	1.22, 1.00	2.10, 1.96	1.82, 1.64
**Self-identified Race/Ethnicity**			
Non-Hispanic White	1.00, Referent	1.00, Referent	1.00, Referent
Non-Hispanic Black	1.61, 1.44	1.40, 1.20	1.44, 1.22

© 2024 Wadhwa A et al. *JAMA Network Open*

eTable 4. Characteristics of the California Cancer Registry Cohort Matched to Greater Los Angeles Veterans Health Administration on Age, Race, Diagnosis Year, Stage, and Census Tract Stratified by Race and Ethnicity (n=2,914)

	Overall	NHW	NHB	Oth	*P*[a]
**n**	2914	1418	1155	341	
Age at diagnosis [mean (SD)]	65.17 (6.71)	65.70 (6.78)	63.96 (6.65)	67.05 (5.89)	<0.001
Age category (years, %)					<0.001
<50	13 (0.4)	<11[d]	11 (1.0)	0 (0.0)	
51-60	653 (22.4)	300 (21.2)	324 (28.1)	29 (8.5)	
61-70	1755 (60.2)	832 (58.7)	682 (59.0)	241 (70.7)	
71-79	471 (16.2)	272 (19.2)	132 (11.4)	67 (19.6)	
80+	22 (0.8)	12 (0.8)	<11[d]	<11[d]	
Diagnosis years (%)					0.005
2000-2004	1183 (40.6)	609 (42.9)	426 (36.9)	148 (43.4)	
2005-2009	714 (24.5)	327 (23.1)	319 (27.6)	68 (19.9)	
2010-2015	896 (30.7)	433 (30.5)	356 (30.8)	107 (31.4)	
2016-2018	121 (4.2)	49 (3.5)	54 (4.7)	18 (5.3)	
Stage at diagnosis (%)					0.342
Localized	2397 (82.3)	1150 (81.1)	967 (83.7)	280 (82.1)	
Regional	508 (17.4)	265 (18.7)	183 (15.8)	60 (17.6)	
Distant	<11[d]	<11[d]	<11[d]	<11[d]	
Follow-up months (median [IQR])	100.67 [55.92, 120.00]	102.64 [58.50, 120.00]	97.02 [53.13, 120.00]	101.46 [52.80, 120.00]	0.085[b]
Deaths from any cause (%)	612 (21.0)	298 (21.0)	259 (22.4)	55 (16.1)	0.043
Prostate cancer (%)	121 (19.8)	46 (15.2)	61 (23.6)	14 (25.5)	0.036
nSES Index (mean (SD))	-0.08 (3.60)	0.52 (4.34)	-0.48 (2.55)	-1.21 (2.72)	<0.001
nSES Quintiles (%)					<0.001
Q1	559 (19.2)	253 (17.8)	196 (17.0)	110 (32.3)	
Q2	595 (20.4)	149 (10.5)	356 (30.8)	90 (26.4)	
Q3	462 (15.9)	203 (14.3)	212 (18.4)	47 (13.8)	
Q4	645 (22.1)	355 (25.0)	225 (19.5)	65 (19.1)	
Q5	653 (22.4)	458 (32.3)	166 (14.4)	29 (8.5)	
Population density ≥1000 people/mi^2 (%)	2549 (87.5)	1093 (77.1)	1147 (99.3)	309 (90.6)	<0.001

[a]Chi-squared test of independence, [b]Wilcoxon Rank Sum Test, [c]Independent two-sample t-test, (% or SD), [d]Cell counts and percentages suppressed for confidentiality [range]. Percentages may not sum to 100 due to rounding.

© 2024 Wadhwa A et al. *JAMA Network Open*

eTable 5. **Multivariable Analysis for Associations of Race and Ethnicity with Prostate Cancer Outcomes in the Veterans Health Administration Cohort and Fully Matched (Age, Race, Diagnosis Year, Stage, Census Tract) California Cancer Registry Cohort**

	Non-Hispanic White	Non-Hispanic Black	P_{het}[f]	Other	P_{het}[f]
**VA Cohort**					
Advanced/Localized (n/n)	35/659	47/786		19/335	
De Novo Metastasis[a]	Ref	1.19 (0.72, 1.96)		1.07 (0.59, 1.92)	
Deaths/Person-years	340/6179	351/7357		157/3120	
All-cause Mortality[b]	Ref	0.90 (0.76, 1.06)		0.85 (0.70, 1.03)	
Deaths/Person-years	35/6179	54/7357		24/3120	
Prostate Cancer-Specific Mortality[c]	Ref	1.13 (0.71, 1.80)		1.36 (0.79, 2.34)	
**CCR Fully Matched[d]**					
Advanced/Localized (n/n)	268/1150	188/967		61/280	
De Novo Metastasis[a]	Ref	0.93 (0.73, 1.18)	0.37	1.08 (0.78, 1.49)	0.97
Deaths/Person-years	298/10375	259/8135		55/2442	
All-cause mortality[b]	Ref	1.10 (0.90, 1.34)	0.12	0.62 (0.46, 0.84)	0.087
Deaths/Person-years	46/10375	61/8135		14/2442	
Prostate Cancer-Specific Mortality[c]	Ref	1.52 (0.98, 2.36)	0.37	1.14 (0.63, 2.06)	0.66

[a]Odd ratios from Multivariable logistic regression analysis for de novo metastasis incidence, [b]Hazard ratios from Cox-Proportional Hazard Model for All-Cause Mortality was performed in the VA and CCR cohorts, [c]Subdistribution hazard ratios from Fine-Gray Analysis for Prostate Cancer Specific Mortality and multivariate logistic regression analysis for de novo metastasis incidence were performed in the VA and CCR cohorts. [e]Matched on age, race, state at diagnosis, year of diagnosis, and census tract. [f]P-value from q-statistic with 1 degree of freedom comparing heterogeneity of association in CCR Fully Matched cohort to GLA cohort. All analyses included the covariates of race, nSES at census tract level (quintiles), age at diagnosis, and year of diagnosis, urbanicity. Models for mortality further included stage at diagnosis. (SIRE = self-identified race/ethnicity, NHW = Non-Hispanic White, NHB/AA = Non-Hispanic Black, Other/Unk = All Other/Unknown, VA = Veterans Administration Cohort, CCR = California Cancer Registry Cohort)

© 2024 Wadhwa A et al. *JAMA Network Open*

eTable 6. Multivariable Analysis for Associations of Neighborhood Socioeconomic Status with Prostate Cancer Outcomes in the Veterans Health Administration Cohort and Fully Matched (Age, Race, Diagnosis Year, Stage, Census Tract) California Cancer Registry Cohort

	Continuous[a]	Quintile 1	Quintile 2	Quintile 3	Quintile 4	Quintile 5	P_{trend}	P_{het}[f]
**VA Cohort**								
Advanced/Localized		15/265	19/396	20/68	27/360	20/391		
[b]De Novo Metastasis	0.90 (0.72, 1.13)	Ref	1.14 (0.55, 2.36)	1.01 (0.50, 2.05)	1.25 (0.64, 2.44)	0.74 (0.37,1.51)	0.31	
Deaths/Person-years		137/2471	198/3751	174/3422	164/3378	175/3631		
[c]All-cause mortality	0.95 (0.87, 1.04)	Ref	0.88 (0.71, 1.10)	0.89 (0.71, 1.12)	0.83 (0.66, 1.04)	0.87 (0.70, 1.10)	0.24	
Deaths/Person-years		14/2471	31/3751	19/3422	28/3378	21/3631		
[d]Prostate Cancer-Specific Mortality	1.23 (0.95, 1.58)	Ref	1.70 (0.89, 3.26)	0.98 (1.02, 0.48)	1.51 (0.78, 2.91)	1.46 (0.73, 2.93)	0.50	
**CCR Fully Matched[e]**								
Advanced/Localized		90/469	87/508	83/379	121/524	136/517		
[b]De Novo Metastasis	1.11 (0.99, 1.25)	Ref	1.06 (0.76, 1.48)	1.21 (0.86, 1.69)	1.32 (0.96, 1.81)	1.29 (0.94, 1.76)	0.080	0.11
Deaths/Person-years		141/4038	134/4505	88/3282	159/4454	90/4673		
[c]All-cause mortality	0.78 (0.71, 0.86)	Ref	0.81 (0.63, 1.03)	0.69 (0.52, 0.91)	0.85 (0.67, 1.08)	0.49 (0.37, 0.66)	<.0001	0.096
Deaths/Person-years		26/4038	29/4505	18/3282	31/4454	17/4673		
[d]Prostate Cancer-Specific Mortality	0.91 (0.72, 1.13)	Ref	0.99 (0.57, 1.73)	0.89 (0.49, 1.63)	1.12 (0.64, 1.94)	0.70 (0.36, 1.35)	0.49	0.0052

[a]Per Interquartile Range, [b]Odds ratios from Multivariable logistic regression analysis for de novo metastasis incidence were performed in the VA and CCR cohorts, [c]Hazard ratios from Cox-Proportional Hazard Model for All-Cause Mortality was performed in the VA and CCR cohorts, [d]Subdistribution hazard ratios from Fine-Gray Analysis for Prostate Cancer Specific Mortality, [e]Matched on age, race, state at diagnosis, year of diagnosis, and census tract. [f]P-value from q-statistic with 1 degree of freedom comparing heterogeneity of association in CCR Fully Matched cohort to GLA cohort.

All analyses included the covariates of race, nSES at census tract level, age at diagnosis, and year of diagnosis, urbanicity. Models for mortality further included stage at diagnosis. Abbreviations: SIRE = self-identified race/ethnicity, NHW = Non-Hispanic White, NHB/AA = Non-Hispanic Black, Other/Unk = All Other/Unknown, VA = Veterans Administration Cohort, CCR = California Cancer Registry Cohort)

© 2024 Wadhwa A et al. *JAMA Network Open*

eFigure 1. Study Flow Diagrams for (A) Greater Los Angeles Veterans Administration and (BC) California Cancer Registry census tract-matched cohorts

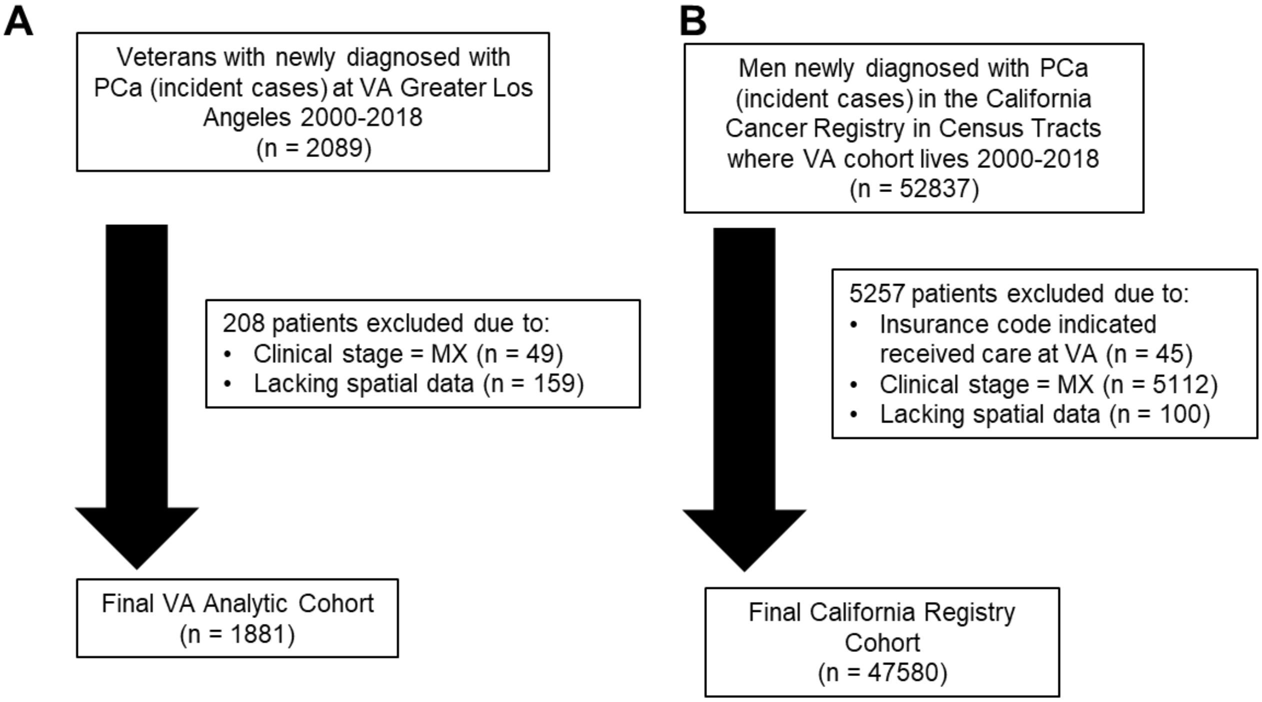

© 2024 Wadhwa A et al. *JAMA Network Open*

eFigure 2. Distribution of Census Tract Socioeconomic Measures in California compared to the Census Tracts in the Greater Los Angeles Veterans Health Administration

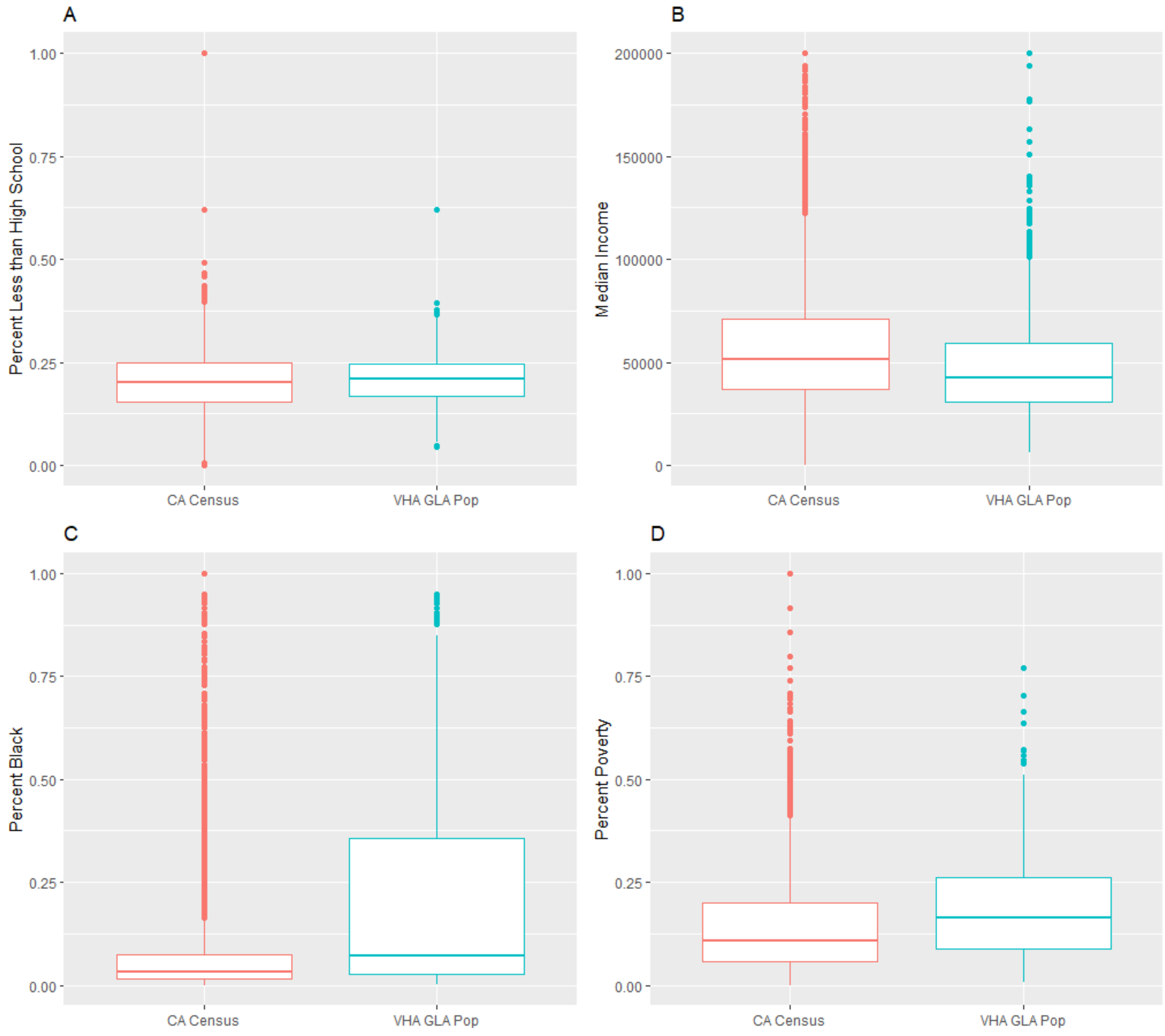

© 2024 Wadhwa A et al. *JAMA Network Open*
