# Supplementary material for: Neighborhood Deprivation, Race and Ethnicity, and Prostate Cancer Outcomes Across California Health Care Systems
Source: JAMA Netw Open. 2024 Mar 19;7(3):e242852. doi: 10.1001/jamanetworkopen.2024.2852 (PMC10951732; doi:10.1001/jamanetworkopen.2024.2852)
Supplement: Supplement 2. — Data Sharing Statement [file jamanetwopen-e242852-s002.pdf]

Data Sharing Statement

Wadhwa. Neighborhood Deprivation, Race and Ethnicity, and Prostate Cancer Outcomes Across California Health Care Systems. *JAMA Netw Open*. Published March 19, 2024. doi:10.1001/jamanetworkopen.2024.2852

Data
Data available: No

Additional Information
Explanation for why data not available: The cancer registries and the VA prohibit individual level data sharing without IRB approval. Anyone interested in obtaining individual level data can contact the corresponding author to discuss how to obtain IRB approval on a case by case basis.
